# Association between successful smoking cessation and preferred smoking time

**DOI:** 10.18332/tid/152413

**Published:** 2022-11-11

**Authors:** Yasuhiro Hashimoto, Akiko Higashiyama

**Affiliations:** 1 Department of Health Sports Communication, Kobe University of Welfare, Fukusaki Town, Japan; 2 Faculty of Sustainable System Sciences, Osaka Metropolitan University, Osaka City, Japan; 3 Faculty of Public Affairs, Osaka University of Commerce, Higashi Osaka City, Japan; 4 School of Health Sciences, Kio University, Koryo, Japan

**Keywords:** smoking cessation support, prevention of smoking relapse, after-meal smoking, after-dinner smoking, smoking cravings

## Abstract

**INTRODUCTION:**

We examined the relationship between preferred daily smoking times and typical situations in which smoking occurs and aimed to determine the association between successful smoking cessation and preferred smoking time.

**METHODS:**

We conducted an internet survey and categorized participants based on their selected smoking status: ‘successful smoking cessation’, ‘failed smoking cessation’, or ‘currently smoking’. Ultimately, 3637 people (1854 men, 1789 women) aged 30–59 years were included in the study. Participants also described the time points at which smoking seemed to be the most appealing and those at which smoking seemed the most difficult to resist.

**RESULTS:**

Regarding times of tobacco cravings, the number of non-smokers for more than 1 year who chose ‘after dinner’ was significantly higher than the number who chose ‘after breakfast’ or ‘after waking up’. Regarding the time when smoking was the most difficult to resist, the proportion of people who chose ‘after dinner’ that had quit smoking for less than 3 months was significantly low.

**CONCLUSIONS:**

Those who prefer smoking ‘after dinner’ are less likely to start smoking cessation, but when they do, the rate of continuation for more than 1 year is high. We suggest that smoking cessation support based on preferred smoking times may lead to a decrease in the smoking rate.

## INTRODUCTION

Smokers start smoking due to various environmental and social factors. In the behavior change model for smoking cessation^1,2^, the stages of continuous smoking cessation are classified into five periods, and intervention methods for each period have been proposed^3-5^. In contrast, Yong et al.^6^ advocated that there are three periods from the beginning of smoking cessation to continuous smoking cessation: 1) an initial implementation period that may last several days; 2) a period of consolidation where there is a restored need for active self-regulation; and 3) a synthesis period where refraining from smoking stops being the primary focal activity. Nicotine dependence^7,8^, motivation^8,9^, feelings of self-efficacy^10^, social environment^11^, and the presence or absence of a smoking cessation program and support from others^12,13^, are factors that contribute to smoking relapse after starting smoking cessation. A wide variety of factors, specifically the number of cigarettes smoked and the presence or absence of friends when smoking, increased the risk of smoking relapse.

Higasiyama et al.^14^ reported that the number of cigarettes smoked is related to daily events, and as the habit develops, people begin to smoke during specific daily events, such as when waking up. In addition, the relationship between meal and cigarette is deep, as the term 'after-dinner cigarette' is widely known^15-17^. Thus, it seems there is a relationship between smoking and daily life events. Cosci et al.^18,19^ proposed smoking may be done habitually not only because of nicotine addiction, but also psychological dependence. With this assertion, smoking behavior is thought to be reinforced by classical conditioning by repeating the behavior at the same time as certain daily life events. Hence the more severe the smoking tendency, the more likely the smoker is to smoke during the event rather than simply experiencing the desire to smoke.

Many previous studies focused on the relationship between events, such as waking up and smoking, as many heavy smokers want to smoke immediately after waking up due to blood nicotine levels dropping while they slept^20-22^. The Fagerström Test for Nicotine Dependence (FTND)^20-23^ and the Heaviness of Smoking Index (HSI)^24,25^ are tools used to measure the degree of dependence. The HSI considers how many minutes after waking up smoking occurs and that this is associated with a higher degree of nicotine dependence, making successful smoking cessation more difficult^24^. The reason for this is likely the desire to replenish the nicotine missed while sleeping; however, there are other possibilities that may be relevant to the initiation and continuation of smoking cessation.

This study used three perspectives (cravings, appealing, and difficulty to quit) to capture the psychological appeal of tobacco based on multiple perspectives. We also examined smokers’ preferences as the ‘cravings’ perspective when using tobacco, while the ‘appealing’ perspective seeks to index smoking satisfaction. For example, the feeling of being ‘appealing’ is thought to be based on the brain’s reward system and the instinctive pleasure a person feels from smoking when they are nicotine-deficient^26^. Tobacco contains nicotine and even though it is a toxic substance, the ingestion of nicotine causes the excessive release of neurotransmitters, such as dopamine and β-endorphin, thereby activating the reward system in the brain^27^. Data show that animals will come to like the taste of something if it is combined with something pleasurable (e.g. morphine is bitter and rats do not prefer morphine over water, but rats that have been addicted to morphine will come to choose morphine over water because of the associated pleasurable feelings)^28^. Based on this logic, we considered the ‘appealing’ variable to be the time when the most pleasure is obtained by smoking. However, the viewpoint of ‘difficult to quit’ was to measure the degree of dependence. Studies show dependence on tobacco is related to time of smoking during the day, as well as blood nicotine levels, such as the inability to refrain from smoking immediately after waking up^29^. ‘Difficult to quit’ is the time when dependence on tobacco is the greatest. When the percentage of smokers who choose this time is high, it is considered strongly dependent. In a previous study^14^, heavy (HSI index of 5–6 points) and medium smokers (HSI index of 2–4 points) were more difficult to quit smoking when waking up than low smokers (HSI index of 0–1 points). However, the previous research focused on waking up, it is considered an event based on the theoretical background of compensating for the deficiency of nicotine. To the best of our knowledge, no research examined the daytime event according to the psychological aspect. Therefore, this study aimed to examine the relationship between smoking and daytime events, focusing on the psychological aspect.

## METHODS

### Participants and study design

The study conducted an online survey in March 2016 and included Japanese people aged 30–59 years who were registered in the Questant system (Macromill, Inc.; Tokyo). In Japan, smoking is allowed for people aged ≥20 years. Since the number of successful quitters is very small for those aged 20–29 years, this survey targeted those aged ≥30 years.

### Measures

The survey controlled the number of participants in the form of aborting the submission of responses at the time when the planned number of participants was reached for each attribute. The attributes at this time included eight residential areas, age (30–39, 40–49, and 50–59 years), sex (male and female), and smoking status. Participants selected their smoking status as ‘never smoked’, ‘successful smoking cessation’ (formerly smoked but not smoking now), ‘smoking cessation failure’ (smoking cessation attempted but failed), and ‘currently smoking’ (never attempted smoking cessation), which were used as the primary dependent variables. Those who selected ‘successful smoking cessation’ were asked to indicate the period of smoking cessation (13 levels).

The independent variables included age, sex, smoking status, and period of smoking cessation. In addition, we also asked four questions to better understand the characteristics of the subjects: ‘How long have you been smoking tobacco?’ (8 levels), ‘How often do you currently crave smoking tobacco? (7 levels), ‘How often do you smoke tobacco?’ (8 levels), and ‘How many cigarettes do you smoke per day?’ (5 levels).

Further, we asked current smokers which daily life events (‘after waking up’, ‘after breakfast’, ‘commuting to school and other places’, ‘after lunch’, ‘during break at work’, ‘after dinner’, ‘when relaxing at home’, ‘after bathing’, ‘before bedtime’, and ‘other’) were associated with cravings (multiple answers allowed). We also asked when smoking seemed to be the most appealing (a single answer) and when smoking seemed the most difficult to quit (a single answer). If the answer to the question was ‘other’, the response category was an open-ended item asking participants to freely write in a description. All procedures used in this study were approved by the Ethical Committee of Kio University and were conducted in accordance with the Declaration of Helsinki. All participants provided consent to participate in this study when completing the online survey.

### Procedure

The target number of responses was ≥4000. Among them, the upper limit was set to maintain equal numbers of age and sex/gender. For smoking experience, we set a higher upper limit than ‘successful smoking cessation’ and ‘smoking cessation failure’ to make a comparison during the non-smoking period in ‘currently smoking’. In addition, the Questant system automatically determines that an answer is invalid despite very limited time to answer. All research in this study was conducted using the Questant system. First, we submitted the survey items to the company and then determined the maximum number of respondents. Finally, the data were collected according to the Micromill, Inc criteria.

The definition of successful quitting duration remains unclear. This may be because it takes only one cigarette for a quitter to smoke again, regardless of the quitting period. Therefore, a wide variety of successful quitting periods exists but very few studies have examined each period. For example, DiPiazza and Naegle^30^ reported that it is challenging to obtain data on quitters for more than a year. Therefore, in this study, the upper limit of the successful quitting period was 1 year, and the period was <3 months, 3 months to <1 year, or ≥1 year. First, we classified them into three categories and distributed the data according to each label. As a result, the smoking statistics reached 5 levels of ‘currently smoking’ (hereinafter, Smoking), ‘smoking cessation failure’ (hereinafter, Failure), and ‘successful smoking cessation’ (hereinafter, Smoking cessation) of 3 levels (‘<3 months’, ‘3 months to <1 year’, and ‘≥1 year’).

### Statistical analysis

The daily life events and three factors of ‘craving’, ‘appealing’, and ‘difficult to quit’ were analyzed using the descriptive statistic of cross tabulation. In the cross tabulation, a chi-squared test and residual analysis were performed when the two variables were on a nominal scale. Inferential statistics were used to measure the ‘craving’ times; these were aggregated for each smoking status, then subjected to one-way analysis of variance. The significance level was set at p<0.05. For this reason, in the residual analysis after the chi-squared test, the adjusted coefficient (±1.96) was used as a criterion of significant difference. SPSS software Ver. 26 for Windows (IBM Corp.; Armonk, NY, USA) was used for the statistical analysis.

## RESULTS

Table 1 shows descriptive statistics on age, gender, smoking experience, and condition of participants. A total of 3637 participants (1854 men, 1789 women) aged 30–59 years were included. The average age was 44.10±8.34 years.

**Table 1:** Descriptive statistics on participants’ age, gender, smoking experience, and condition (N=3637)

*Characteristics*	*%*
**Age at recruitment** (years)	
30–39	33.98
40–49	33.98
50–59	32.03
**Sex**, male	50.98
**Smoking status**	
Smoking	9.79
Failure	7.20
Smoking cessation	
<3 months	15.59
3 months to <1 year	16.44
≥1 year	50.98
**How long have you been smoking tobacco?** (years)*	
<1	4.87
1–2	6.52
3–5	5.75
5–10	12.29
10–15	14.98
15–20	14.85
>20	38.33
Do not know	2.42
**How often do you currently crave smoking tobacco?**	
Always want to smoke	6.41
Want to smoke more than once a day	19.55
Want to smoke about once a day	5.42
Want to smoke about once every 2 or 3 days	3.63
Want to smoke about once a week	3.66
Sometimes I want to smoke, but more often	27.91
Do not want to smoke at all	33.43
How often do you smoke tobacco?*	
Every day	76.93
2 or 3 days a week	4.23
4 or 5 days a week	1.10
Once a week	1.76
Once every few weeks	0.74
Once a month	0.93
Once every few months	1.04
Less frequently	13.25
How many cigarettes do you smoke per day?*	
<10	43.91
10–19	40.20
20–29	10.78
30–40	3.19
≥41	1.92

* If not currently smoking, participants were asked to base their answers on the time they were smoking. These data were acquired in Japan in March 2016.

Table 2 shows the various times the participants had cravings during the day. The average time was 5.53±2.55 for smokers; 4.70±2.46 for participants who quit smoking; 4.71±2.72 for non-smokers who quit for <3 months; 4.62±2.71 for those who have quit for ≥3 months but less than a year; and 4.79±2.76 for those with continued cessation for ≥1 year. In the analysis of variance results, the main effect of smoking status was observed (F [4, 3636]=7.28, p<0.001) in smokers as a result of multiple comparisons: failed smoking cessation; smokers who have quit for <3 months and <1 year; and continuous smoking cessation. The number of cravings was significantly greater in those with <1 year cessation than in those who continued for ≥1 year.

**Table 2:** Cross tabulation of smoke craving times and status

		*After waking up*	*After breakfast*	*Commuting*	*After lunch*	*During break at work*	*After dinner*	*When relaxing at home*	*After bathing*	*Before bedtime*	*Other*	*Average number of choices*	*n*
**Smoking**		64.04**	60.11**	36.52**	77.53**	66.85	75.84**	73.60**	41.85**	55.90**	0.56**	5.53	356
		(5.39)	(2.83)	(2.98)	(3.38)	(1.63)	(2.59)	(2.76)	(4.71)	(4.44)	(-3.17)	(2.55)	
**Failure**		51.15	58.78	24.43	67.56	54.20**	70.61	67.56	30.15	45.04	0.76*	4.70	262
		(0.22)	(1.94)	(-1.93)	(-0.78)	(-3.02)	(0.27)	(0.18)	(-0.27)	(0.08)	(-2.50)	(2.46)	
**Smoking cessation**	<3 months	49.56	49.03*	30.16	68.96	61.90	66.67	64.37	29.81	45.15	5.64**	4.71	567
		(-0.48)	(-2.07)	(0.28)	(-0.42)	(-0.52)	(-1.81)	(-1.48)	(-0.62)	(0.19)	(3.04)	(2.72)	
	3 months to <1 year	45.99*	52.17	28.93	66.56	60.70	68.23	65.38	28.43	43.14	2.84	4.62	598
		(2.41)	(0.45)	(0.43)	(1.83)	(1.21)	(0.96)	(0.95)	(1.43)	(0.89)	(0.95)	(2.71)	
	≥1 year	49.51	52.32	29.18	69.74	64.35	70.12	67.10	30.04	43.04	3.99	4.79	1854
		(-1.19)	(-0.85)	(-0.66)	(0.06)	(1.87)	(0.34)	(0.05)	(-1.15)	(-2.16)	(1.67)	(2.76)	
**Total**		50.48	53.01	29.67	69.70	62.88	69.87	67.06	30.90	44.79	3.49	4.82	3637

* p<0.05.

** p<0.01.

The upper value is the ratio of ‘After waking up’ to ‘Other’, while the lower value in brackets shows the adjusted coefficient in the residual analysis. ‘Average number of choices’ indicates the mean number with standard deviation in brackets. The green shaded area in the table indicates significantly higher frequency. The yellow shaded area in the table indicates significantly lower frequency. These data were acquired in Japan in March 2016.

Table 3 shows the time tobacco is most ‘appealing’ during the day. The chi-square test revealed significant differences in which a daily life event was considered the most appealing event (χ^2^ [36]=63.70, p=0.003). Further, multiple analysis results showed the selectivity rate for ‘after breakfast’ for smoking cessation failure, ‘other’ for ≥1 year or <3 months, and ‘after dinner’ for ≥1 year was significantly higher. In contrast, the selectivity rate was significantly lower for ‘other’ for ≥1 year, ‘during a break at work’ by smoking cessation failure, and ‘after breakfast’ for ≥1 year.

**Table 3:** Cross tabulation of the most ‘appealing’ smoking times and smoking status

		*After waking up*	*After breakfast*	*Commuting*	*After lunch*	*During break at work*	*After dinner*	*When relaxing at home*	*After bathing*	*Before bedtime*	*Other*	*Total*
**Smoking**		43	23	7	62	53	98	59	3	7	1**	356
		(0.07)	(1.93)	(-0.35)	(1.24)	(0.02)	(0.04)	(-0.48)	(0.17)	(-0.35)	(-3.43)	
**Failure**		35	20**	7	38	24**	72	54	1	5	6	262
		(0.72)	(2.59)	(0.51)	(-0.32)	(-2.69)	(0.02)	(1.38)	(-0.75)	(-0.36)	(-1.04)	
**Smoking cessation**	<3 months	66	31	17	90	87	143	87	3	12	31**	567
		(-0.26)	(1.27)	(1.35)	(0.50)	(0.36)	(-1.29)	(-1.46)	(-0.71)	(-0.19)	(2.94)	
	3 months to <1 year	72	29	17	85	90	147	116	8	17	17	598
		(0.07)	(0.51)	(1.12)	(-0.72)	(0.15)	(-1.71)	(1.35)	(1.74)	(1.12)	(-0.84)	
	≥1 year	219	59**	33	277	286	538*	320	13	40	69	1854
		(-0.28)	(-3.79)	(-1.86)	(-0.41)	(1.00)	(2.18)	(-0.37)	(-0.48)	(-0.29)	(-0.30)	
**Total**		435	162	81	552	540	998	636	28	81	124	3637

* p<0.05;

** p<0.01.

The upper value is the ratio while the lower value in brackets shows the adjusted coefficient in the residual analysis. The green shaded area in the table indicates significantly higher frequency. The yellow shaded area indicates significantly lower frequency. These data were acquired in Japan in March 2016.

Table 4 shows the times during the day that are most ‘difficult to quit’. The chi-squared test revealed significant differences in which a daily life event was considered the most difficult to quit (χ^2^ [36]=56.26, p=0.017). Further, multiple analysis results showed that the selectivity rate for ‘after waking up’ for current smokers and ‘after dinner’ for ≥1 year was significantly higher. In contrast, the selectivity rate was significantly lower for ‘other’ by current smokers, ‘after dinner’ for <3 months, and ‘after waking up’ for ≥1 year.

**Table 4:** Cross-tabulation of the most ‘difficult to quit’ smoking time and smoking status

		*After waking up*	*After breakfast*	*Commuting*	*After lunch*	*During break at work*	*After dinner*	*When relaxing at home*	*After bathing*	*Before bedtime*	*Other*	*Total*
**Smoking**		65**	21	7	38	59	74	68	0	19	5**	356
		(2.78)	(1.55)	(-1.07)	(0.13)	(-0.42)	(-0.66)	(-0.73)	(-1.55)	(1.47)	(-2.79)	
**Failure**		41	17	10	25	38	59	53	1	12	6	262
		(1.15)	(0.57)	(1.04)	(0.69)	(0.30)	(-2.49)	(0.03)	(0.34)	(-1.92)	(1.81)	
**Smoking cessation**	<3 months	85	27	20	64	101	103*	117	4	14	32	567
		(1.15)	(0.57)	(1.04)	(0.69)	(0.30)	(-2.49)	(0.03)	(0.34)	(-1.92)	(1.81)	
	3 months to <1 year	76	22	20	68	109	120	128	5	26	24	598
		(-0.60)	(-0.84)	(0.78)	(0.78)	(0.60)	(-1.35)	(0.54)	(0.80)	(0.61)	(-0.29)	
	≥1 year	223**	70	47	186	325	450**	383	12	71	87	1854
		(-2.60)	(-1.64)	(-1.20)	(-0.89)	(0.25)	(3.13)	(0.10)	(0.34)	(-0.24)	(1.40)	
**Total**		490	157	104	381	632	806	749	22	142	154	3637

* p<0.05;

** p<0.01.

The upper value is the ratio while the lower value in brackets shows the adjusted coefficient in the residual analysis. The green shaded area in the table indicates significantly higher frequency. The yellow shaded area indicates significantly lower frequency. These data were acquired in Japan in March 2016.

## DISCUSSION

We examined the relationship between daily life events and smoking status based on three factors: craving, appealing, and the most difficult to quit. Our results indicate current smokers had a higher selection rate for ‘craving’ on each event (‘after breakfast’, ‘commuting’, ‘after lunch’, ‘after dinner’, ‘after bathing’, ‘before bedtime’, and ‘after waking up’) than those with successful smoking cessation or smoking cessation failure. From this, we can assume smokers wanted to smoke tobacco at various times of the day besides ‘after waking up’, as pointed out in previous research, and as compared to those with failed smoking cessation and people who have quit smoking. Smokers often have a higher selection rate than those who do not currently smoke. Therefore, it is considered that the experience of smoking cessation indicates a transition to a different stage of smoking and is a step towards quitting, regardless of success or failure, as current smokers typically want to smoke all the time whereas smokers who have tried smoking cessation do not. At the same time, this result shows the preparatory period and the execution period in the transtheoretical model^3^ are supported even when the preference for smoking time is examined. In addition, since the ‘other’ selection rate was 3.49% on average for all the participants, it is considered that the selection was appropriate because there were few events that wanted to smoke other than the items used in this survey.

Current smokers chose ‘after waking up’ as a ‘difficult to quit’ more than the other groups; however, this category was chosen less by those with continuous smoking cessation for over 1 year. This result supports the prerequisites of the FTND index^21-22^ and the HSI^23,24^, which reported the correlation between nicotine dependence and preferred smoking time. However, there was no difference between the groups for when smoking was most appealing. For all smokers, ‘after waking up’ smoking time was considered to be the most ‘difficult to quit’ and the most ‘appealing’ time.

Respondents who failed with smoking cessation chose ‘after breakfast’ as the ‘difficult to quit’ time, as did a few smokers who quit for more than 1 year. Whether those with failed smoking cessation think ‘after breakfast’ is an appealing time to smoke may be a factor in predicting the success or failure of smoking cessation. The ‘smoking cessation for more than 1 year’ group included many who chose ‘after dinner’ as the time when tobacco was ‘appealing’ or ‘difficult to quit’. Previous studies have shown using the FTND index that the appeal of smoking after waking up affects the success or failure of quitting^20^. However, the results of our study showed that whether the participant wanted to smoke ‘after breakfast’ rather than ‘after waking up’ may be more accurate in predicting the success or failure of quitting. It is possible that those with failed smoking cessation do not think that smoking in the morning is needed to satisfy nicotine cravings, but they may consider it a nutritional supplement to their breakfast. Therefore, emphasizing and disseminating the perception that nicotine is a poison rather than nutritional may be effective for those who struggle to quit smoking.

Non-smokers for <3 months had low selectivity of ‘after dinner’ as ‘difficult to quit’. From these facts, it is difficult for smokers who prefer ‘after dinner’ as a smoking time to initiate smoking cessation, although the results suggest that when they stop smoking, there is a high possibility of continuing it for ≥1 year. In this study, we found that smokers have a habit of smoking after meals during the day. This may be the norm for many, but no study has reported this hitherto. Furthermore, the participants stated that after meals, it was ‘appealing’ and ‘difficult to quit’ smoking compared to the urge during ‘commuting’ and ‘before bedtime’. These findings suggest that the time after a meal (breakfast or dinner) is an important time when smokers consider to smoke. However, since nicotine deficiency does not necessarily occur after dinner, it is presumed that smoking occurs because it is psychologically ‘appealing’. This is supported by the fact that dinner is not only the most ‘difficult to quit’ time (Table 4) but also the ‘most appealing’ time (Table 3) for more than one year after smoking cessation. Smoking after dinner was the event associated with most cravings (Table 2). Therefore, perhaps smoking after dinner is not due to the smokers’ intention but rather a situation wherein smoking is induced by the event. In other words, smoking after dinner often ingrains the habit of smoking through classical conditioning, and smokers may have to have quit for a long time before being free of the conditioning.

The reason people who prefer ‘after dinner’ as a smoking time have a high rate of smoking cessation is thought to be that they smoke after meals as a habit by operant conditioning^31,32^ rather than the need for nicotine supplementation. In addition, it is presumed that one of the factors with ‘after dinner’ is the short time between smoking and going to sleep, therefore the time when nicotine intake will be cut off is not very far off. However, the number of people who prefer ‘after dinner’ as the smoking time was significantly less among non-smokers of <3 months. The z-value of the residual analysis was negative, even for those who failed in smoking cessation and those who were non-smokers for ≥3 months but <1 year. From these results, we infer that those who prefer ‘after dinner’ as the smoking time have difficulty initiating quitting smoking; however, they are more likely to be successful with long-term smoking cessation. Based on the above, this study suggests asking two questions: 1) ‘Is smoking the most appealing after breakfast?’, and 2) ‘Is after dinner the most difficult time of day to refrain from smoking?’. Answers to these questions are important for designing supportive smoking cessation programs and proposes the need to adjust the interventions depending on the experiences and preferences of smokers. Smokers who answered in the affirmative to the first question were more likely to fail even if they started smoking cessation. For this reason, it is necessary to actively encourage smoking cessation and to include support from others by actively utilizing social support^33^. Smokers who answered ‘yes’ to the second question are likely to continue smoking cessation once started and there is a high probability that they will not start smoking again. Therefore, we suggest smoking cessation programs include information on the merits of smoking cessation and specific methods that can be used to achieve it for a long term. A meta-analysis of smoking cessation reported that smoking cessation support did not have a significant effect on preventing relapse^34^; however, these two questions may reduce the amount of smoking cessation drugs, such as varenicline, required and increase the effectiveness of face-to-face smoking cessation support.

### Strengths and limitations

This study examined factors that continue smoking cessation from a new perspective, daily life events, in a large-scale study sample of 3637 people. The results of the study clarified the relationship between the preference for smoking after dinner and the continuation of smoking cessation. However, due to the limited survey design, it is not possible to clarify why the relationship between the two is significant. This study used the χ^2^ test to determine the relationship between smoking experience and events, examining the highs and lows of their ratio. However, to investigate the direct relationship between each factor, it is necessary to examine it with a regression model^20^. Therefore, it is considered that adding items related to smoking after breakfast and dinner is effective for improving the model’s suitability. Future studies will need to investigate the characteristics of ex-smokers who preferred to smoke after dinner. By examining the dependence on tobacco through the relationship between smoking behavior and daily life events, it is thought that the findings can be utilized for early prevention and treatment. Among them, the degree of stress that the participants have and the person they are eating dinner with. It is also considered effective to ask about their ways of relieving stress.

A strength of this study is that our data were obtained using an online survey by a research company, which allowed the recruitment of a large number of participants. This enabled us to acquire the necessary number of attributes (sex/gender, smoking status, and residential areas) without the need for arbitrary selection by us. It was not realistic to control the samples in individual studies when surveys were conducted by mail using questionnaires (i.e. only a national survey was possible). In this survey, we used an epoch-making method to control the attributes of our participants. However, the more attributes are controlled in a questionnaire survey, the more difficult it is to practice the principle of complete random sampling. Moreover, a detailed research plan is particularly important for accurate measurement; however, the balance examination is still new. In this study, we created questions to understand the characteristics of the included participants. This study showed that it is more important to describe the data characteristics to be analyzed in a wide range online survey than in a questionnaire.

## CONCLUSIONS

We examined the characteristics of successful and unsuccessful smoking cessation based on preferred daily smoking times. A high percentage of those who failed in smoking cessation replied that smoking after breakfast was the most appealing time of the day. A high percentage of non-smokers for ≥3 months but <1 year replied that after dinner was the most difficult time to quit. For these reasons, it is possible to increase the success rate of smoking cessation by asking these two questions when developing supportive smoking cessation programs and adjusting the interventions according to the answers.

## Data Availability

The data supporting this research are available from the authors on reasonable request.
